# Mapping electrochemically driven gas exchange of mixed conducting SrTi_0.7_Fe_0.3_O_3 − δ_ and Ce_0.8_Gd_0.2_O_1.9_ thin films by ^18^O tracer incorporation under reducing atmosphere

**DOI:** 10.1016/j.ssi.2014.10.024

**Published:** 2015-05

**Authors:** Andreas Nenning, Edvinas Navickas, Peter Velicsanyi, Alexander K. Opitz, Herbert Hutter, Jürgen Fleig

**Affiliations:** Vienna University of Technology, Institute of Chemical Technologies and Analytics, Getreidemarkt 9/164, 1060 Vienna, Austria

**Keywords:** Thin film electrode, Isotopic surface exchange, ^18^O enriched water, Electrochemical water splitting, Electrochemically active zone

## Abstract

Thermally and electrochemically driven ^18^O tracer exchange experiments in H_2_/H_2_^18^O atmosphere were performed on SrTi_0.7_Fe_0.3_O_3 − δ_ and Ce_0.8_Gd_0.2_O_2 − δ_ thin films on single crystalline YSZ substrates. Noble metal current collectors were deposited on both films and electrochemically polarized during the exchange experiment. The resulting tracer distribution was analyzed by spatially resolved secondary ion mass spectrometry. Increased tracer fraction near the current collectors was found under cathodic polarization and decreased tracer fraction under anodic polarization. High cathodic bias leads to enhanced n-type electronic conductivity, which increases the extent of the electrochemically active zone.

## Highlights

• ^18^O from tracer marked water was incorporated in SrTi_0.7_Fe_0.3_O_3–δ_ and Ce_0.8_Gd_0.2_O_1.8_ thin films.
• On both MIEC thin films, Pt and Au current collectors were applied beneath the film and ontop, respectively.
• Cathodic polarization extended the oxygen incorporation zone from the current collector along the free electrode surface.

## 1 Introduction

Mixed ionic and electronic conduction (MIEC) is widely investigated for intermediate-temperature solid oxide fuel cell (SOFC) cathodes [1], [2], [3] and there is also strongly growing interest in applying reduction stable MIECs as SOFC anodes [4], [5], [6], [7], [8], [9], [10]. Studies on some perovskite-type porous anodes demonstrated low area specific resistance and high stability for redox cycling [4], [5], [6], [7]. For ceria-based anode materials also mechanistic investigations on geometrically well-defined thin films have been performed [8], [9], [10]. However, details on the surface exchange rate and ionic or electronic conductivity are still scarce for most oxide electrodes in reducing atmosphere. Additional measurements, also employing new analysis methods, are therefore of high relevance for a better understanding of the properties of mixed conducting anodes and the search for new materials.

It has been demonstrated that isotope exchange with subsequent SIMS analysis is a very powerful tool to monitor the surface reaction kinetics and the bulk diffusion of oxygen in SOFC electrode and electrolyte materials under both equilibrium and polarized conditions [11], [12], [13], [14], [15], [16], [17], [18]. Moreover, because of the relatively high lateral and depth resolution of SIMS, the electrochemically active regions of the electrodes can be visualized. This has already been shown for cathode materials [14], [17], while in reducing atmosphere imaging of oxygen incorporation zones by voltage driven ^18^O tracer exchange is rarely employed.

In the present study, we analyze the distribution of ^18^O in polarized and non-polarized thin films of SrTi_0.7_Fe_0.3_O_3 − δ_ (STFO) and Ce_0.8_Gd_0.2_O_2 − δ_ (GDC) on yttria stabilized zirconia (YSZ) substrates. By comparison of the tracer distribution after thermally driven oxygen exchange and after experiments with cathodic and anodic bias, the width of the electrochemically active region and the factors governing the oxygen exchange could be visualized.

## 2 Experimental

### 2.1 Sample preparation

The GDC target (Ce_0.8_Gd_0.2_O_2 − δ_) for pulsed laser deposition was prepared from powder (Treibacher, Austria) by isostatical pressing and subsequent sintering at 1550 °C for 5 h. The STFO (SrTi_0.7_Fe_0.3_O_3 − δ_) powder was prepared by solid state reaction from SrCO_3_ (99.99% pure, Sigma-Aldrich), TiO_2_ (99.99% pure, Sigma Aldrich), and Fe_2_O_3_ (99.98% pure, Sigma Aldrich). The educts were thoroughly mixed, calcined at 800 °C, ground, again calcined at 1000 °C, and—after a further grinding step—isostatically pressed and sintered at 1250 °C. The phase purity of both targets was confirmed by X-ray diffraction. STFO and GDC thin films were deposited on (100)-oriented yttria stabilized zirconia single crystals (YSZ, 9.5 mol% Y_2_O_3_ in ZrO_2_, supplier: CrysTec, Germany) by pulsed laser deposition (PLD) using a KrF excimer-laser (Lambda COMPexPro 201 F, 248 nm wavelength). The deposition of 200 nm thin films was carried out in 4 × 10^− 2^ mbar of pure oxygen with a pulse repetition rate of 5 Hz and a nominal pulse energy of 400 mJ. The substrate temperature during the deposition was controlled by a pyrometer (Heitronics, Germany) and was 650 °C.

### 2.2 Electrode design

**Fig. 1:**
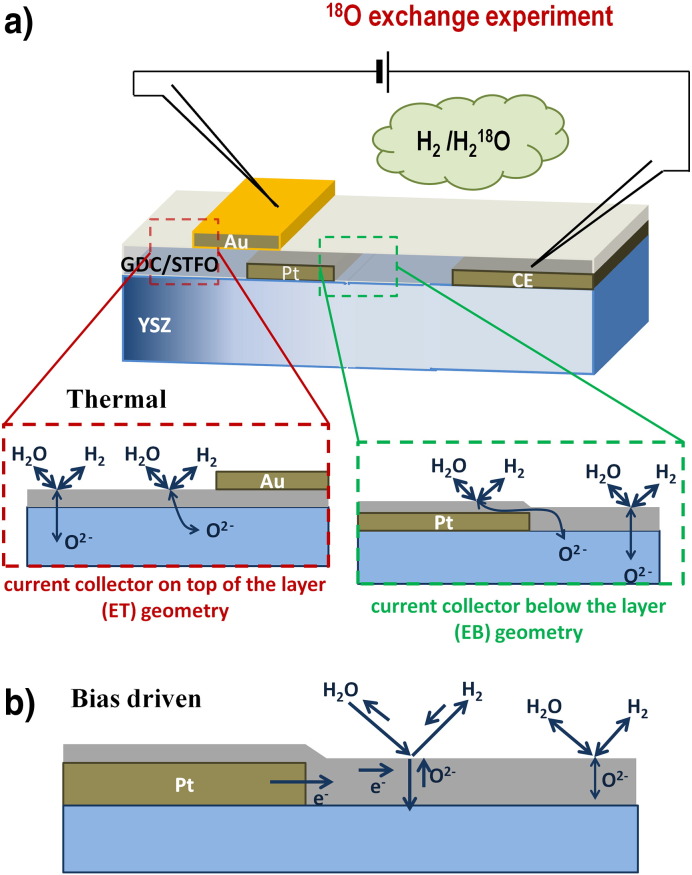
(a) Noble metal electrodes, which provide an electrical contact and block oxygen diffusion were prepared below (EB geometry) and on top (ET geometry) of the GDC or STFO layer. The oxygen diffusion pathways under equilibrium conditions are sketched for ET and EB current collectors. Working and counter electrodes were contacted and polarized in the tracer exchange chamber. (b) Increased oxygen incorporation rate caused by cathodic bias near an EB current collector; in some distance from the current collector edge only thermally driven tracer exchange remains due to limited electronic conductivity within the MIEC thin film.

Acceptor-doped mixed conductors are often very good electronic p-type conductors in oxidizing atmosphere. For such materials, an electric contact with a metallic tip is typically sufficient for a homogeneous polarization of a small thin film electrode [19]. In reducing atmosphere, however, the p-type conductivity decreases by several orders of magnitude. Despite acceptor doping SrTi_0.7_Fe_0.3_O_3 − δ_ (STFO) and Ce_0.8_Gd_0.2_O_1.9_ (GDC) are even weak n-type conductors in reducing atmosphere at the ^18^O exchange temperature [20], [21], [22], [23]. Due to the much lower electronic conductivity compared to oxidizing conditions, the electrochemically active region is expected to be limited to a small area around the electric contact. In order to investigate the width of this active region, rectangular (160 μm × 400 μm) noble metal current collectors were sputter deposited (MCS 020, BAL-TEC AG, Germany) in two steps: on top of the YSZ substrate (prior to MIEC deposition) and on top of the deposited MIEC layer. The sample design is sketched in Fig. 1a; bottom and top current collectors and one large counter-electrode were placed on one and the same sample. The bottom current collectors (5 nm Ti/100 nm Pt) were structured from a thin film by ion beam etching, the top electrodes (5 nm Cr/100 nm Au) were produced by a lift-off method. For the buried current collectors platinum was chosen, since it is sufficiently stable during subsequent PLD deposition of the oxide. For the top electrodes Au was used owing to its poor catalytic activity to avoid three phase boundary activity. For the sake of clarity, the electrode regions with contacting noble metal collectors are named as electrodes with top current collectors (ET) and electrodes with bottom current collectors (EB), and abbreviations ET and EB are used throughout the text.

### 2.3 Procedure of the isotope exchange experiments

A mixture of hydrogen and ^18^O tracer enriched water was used to carry out the experiments in a thermodynamically defined, reducing atmosphere. This atmosphere was produced by mixing diluted hydrogen (2.5% H_2_ in Ar, Alphagaz ARCAL 10, Air Liquide) with a defined amount of ^18^O_2_ (97% isotopic enrichment, 0.625% O_2_ in the mixture) and feeding this mixture through a platinum sponge at 500 °C to form a gas containing equal amounts of water and hydrogen. The formation of tracer-enriched water with a tracer fraction of 60–70% was monitored by a mass spectrometer (Pfeiffer, OmniStar GSD 320). Probably the (porous) quartz supported Pt catalyst, which is present in the reaction chamber, is a source of oxygen exchange and therefore reduces the tracer content in the resulting atmosphere. In this reducing, tracer containing atmosphere, the samples were heated from room temperature to 410 ± 10 °C for 10 min and were subsequently quenched to freeze the distribution of ^18^O (the corresponding oxygen partial pressure in the mentioned humid hydrogen atmosphere at 410 °C can be calculated to be 8.4 × 10^− 30^ bar [24]). The heating and cooling rate was 150 °C/min, so the time of sample heating and cooling was short compared to the exchange experiments. During the exchange process, on each sample one ET and one EB current collector were simultaneously polarized against the counter electrode—cf. Fig. 1a. Current flow and out of equilibrium reaction for ET polarization are sketched in Fig. 1b in more detail. In this manner, the tracer distribution can be monitored in the MIEC above the electrolyte, above the EB current collectors and beneath ET current collectors for the case of pure thermal diffusion as well as for different dc polarization on the same sample.

The resulting ^18^O distribution in the thin films was subsequently investigated by means of time-of-flight secondary ion mass spectrometry (ToF-SIMS). These measurements were done on a ToF-SIMS 5 machine (ION-TOF GmbH, Germany) in collimated burst alignment (CBA) mode, which allows accurate determination of ^18^O concentration in oxides [25], [26]. As primary ions Bi_3_^++^ were used (25 kV accelerating voltage). Negative secondary ions were analyzed in areas of 50 μm × 50 μm and 160 μm × 160 μm, using a raster of 512 × 512 and 1024 × 1024 pixels, respectively. For the sputtering of material Cs^+^ ions (1 kV accelerating voltage) were used with a sputter crater of 500 μm × 500 μm and a sputtering ion current of 70 nA. The charging of the surface was compensated with an electron flood gun and partly by additional argon flooding if the electron flood gun was not sufficient. The isotope fraction (*f_18O_*) was obtained by normalizing integrated intensities (*I)* via *f_18O_ = I_18O_/(I_18O_ + I_16O_)*.

In Fig. 1a, the different locations of the SIMS analysis and the pathways of thermally driven gas exchange are sketched. The metal layers are supposed to be sufficiently blocking for oxygen, which was experimentally confirmed by measuring a tracer fraction close to natural abundance beneath the EB current collector as well as beneath the ET collectors (see Section 3). The remaining slight tracer enrichment beneath the Au current collectors (ET), which is nearly two orders of magnitude lower than without a metal layer, may be due to some grain boundary diffusion of oxygen through the metal layer [13]. Nonetheless, the amount of tracer beneath the current collectors is sufficiently small to assume blocking metal layers in the discussion. In order to compare the lateral isotope distribution of different electrodes, the current collector and MIEC layer boundaries were aligned to the same position in the images.

## 3 Results and discussion

### 3.1 Thermal diffusion profiles

**Fig. 2:**
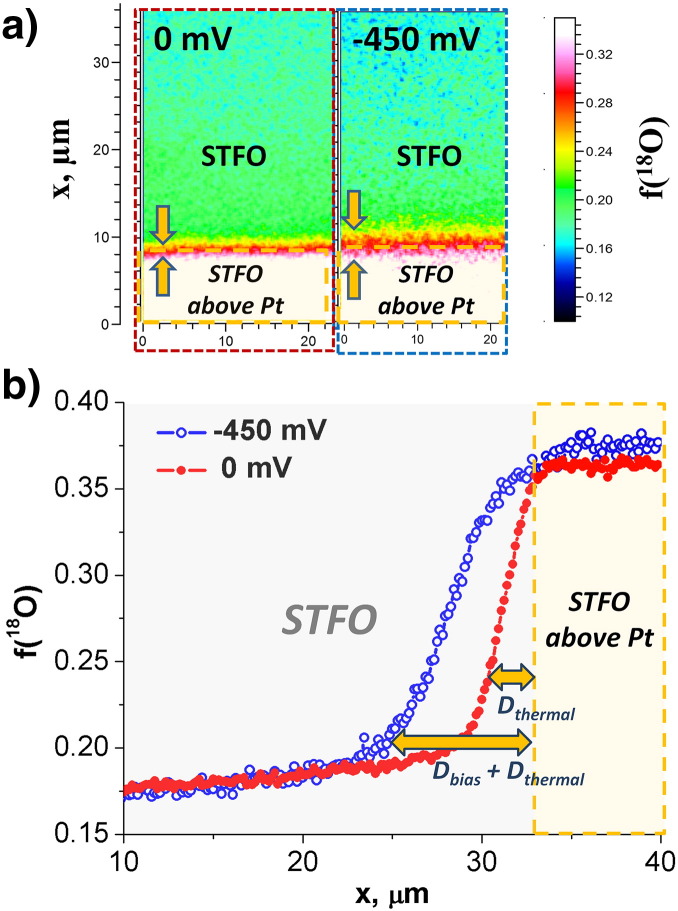
(a) Isotope distribution images measured by ToF-SIMS and (b) lateral tracer fraction profiles of an STFO thin film near to the edge of an electrode with EB geometry for thermally and bias driven ^18^O incorporation.

**Fig. 3:**
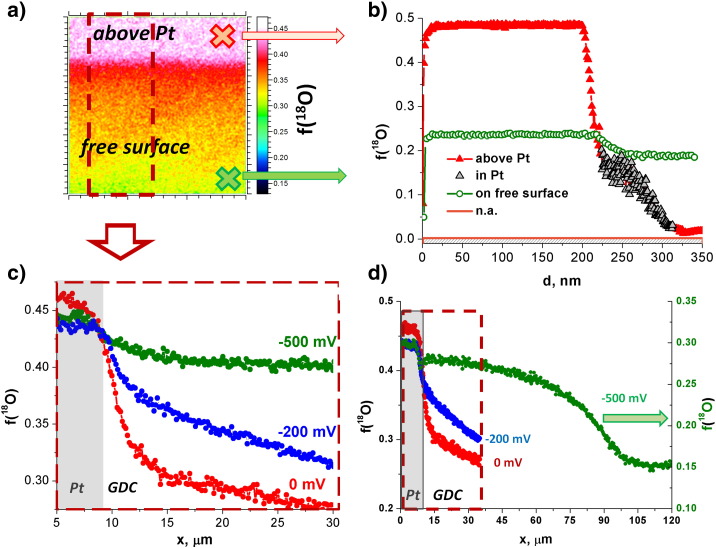
(a) Isotope distribution image with lateral tracer fraction profiles in a GDC thin film near to an EB current collector and the corresponding profiles for three different bias values (c and d). Cathodic bias (− 200 mV: blue curve; − 500 mV: green curve) locally increases the electronic conductivity and thus the width of the electrochemically active zone. (b) The depth profiles of isotope concentration were checked in the MIEC film on top of the EB current collector (red triangles) and on top of YSZ (green circles).

Distribution images of ^18^O as well as lateral profiles of the ^18^O fraction obtained under equilibrium conditions (EB geometry) are depicted in Fig. 2, Fig. 3 for STFO and GDC, respectively (in the lateral profiles the zero-bias curves are given in red color). The zero-bias profiles of both materials exhibit laterally almost constant tracer fractions for the free MIEC part and an increase to a higher value close to the EB current collector edge. The high tracer content above the EB current collectors can be explained by the oxide ion blocking character of the Pt layer, which impedes the diffusion of ^18^O^2−^ ions into the YSZ electrolyte. Therefore, the tracer is simply piled up above Pt, leading to the high tracer fraction observed. Strongly different surface exchange coefficients k^⁎^ caused by different microstructures of the MIEC parts on Pt and YSZ can be ruled out by previous electrochemical experiments [10], [20], [27]. It should further be noted, that the depth profiles reveal constant tracer fraction within the MIEC film, thus indicating surface kinetics to be almost exclusively rate limiting for the thermal exchange of oxygen in both materials (cf. Fig. 3b for GDC; the small concentration step at the MIEC/YSZ interface indicates an additional small resistance).

Interestingly, for both MIEC materials the zero-bias lateral tracer distribution exhibits a certain slope rather than an ideal step close to the edge of the EB current collectors. This lateral profile can be explained by in-plane diffusion of oxygen in the MIEC film from the region with high tracer fraction (above Pt) to the MIEC on YSZ and then into the electrolyte—see Fig. 1a, EB. Hence, the width of this slope should correlate with the diffusion length of oxygen ions. By comparison of Figs. 2b and 3c it becomes obvious that the thermal profile of STFO is steeper than that of the GDC film, which is expected due to the higher ionic conductivity in GDC (σ_GDC_ = 3.3 × 10^− 4^ S cm^− 1^ [21], σ_STFO_ = 5.5 × 10^− 6^ S cm^− 1^ [20] at 410 °C).

### 3.2 Effect of cathodic bias

The incorporation of ^18^O into the region close to the EB current collector changes upon polarization (see Fig. 2, Fig. 3). Close to the current collector, tracer incorporation is enhanced with cathodic bias and the increase of the tracer fraction in the active zone is partly (or even mostly) due to the (electro-) chemically driven current. At a sufficient distance from the electrode, however, the surface exchange rate of ^18^O again approaches its equilibrium value. This can be easily understood by the following consideration: On the one hand, the cathodic voltage causes an increased ^18^O incorporation rate into the MIEC. On the other hand, the electronic sheet resistance in the MIEC becomes more important for longer distances from the current collector and hence causes a decay of the voltage driven ^18^O incorporation rate (this is sketched in Fig. 1b).

Also on top of the Pt current collector, the tracer fraction was found to be virtually unaffected by the applied bias (the slight differences in the tracer fraction on top of the Pt electrodes can probably be attributed to a small inhomogeneity of the surface exchange coefficient e.g. due to a slight temperature inhomogeneity). This is due to the fact that oxygen ions incorporated above the current collector need to diffuse from the reaction site, which is remote from the current collector edges, into the electrolyte. Because of the high in-plane transport resistance of the oxygen ions [20], [21], the MIEC above the current collector remains unpolarized and the tracer fraction is independent of the applied bias. Accordingly, this region is not electrochemically active despite having the highest tracer fraction.

GDC exhibits a larger active zone than STFO upon cathodic bias, which is most probably caused by its higher electronic conductivity (2 ± 0.5 × 10^− 4^ S cm^− 1^ for GDC [21] and 1.5 ± 0.5 × 10^− 5^ S cm^− 1^ for STFO [20] at 410 °C without bias). At higher cathodic bias (− 500 mV) the electrochemically active zone of GDC becomes very broad, see Fig. 3d. (The lower isotope fraction measured in the larger measurement area of 150 μm × 150 μm—shown in Fig. 3c, d—might be caused by longer measurement time together with flooding by Ar gas. Since Ar contains some oxygen residuals this could lead to certain ^16^O coverage on the surface.) At the investigated p(O_2_) of 8.4 × 10^− 30^ bar the well investigated defect model of GDC implies a large, nearly constant number of oxygen vacancies but an electron concentration and thus an electronic conductivity proportional to p(O_2_)^−1/4^ [1], [21]. According to Nernst's equation, a cathodic bias is equivalent to a decrease in oxygen partial pressure and therefore leads to enhanced electronic conductivity in the polarized region, which strongly increases the extent of the electrochemically active zone. Thus, the lateral broadening for increasing voltage can be explained by a polarization driven increase in electronic conductivity. Similar results were obtained for Pt electrodes on YSZ, where a broadening of the active zone for a very high cathodic bias was shown in oxygen atmosphere [14]. There, YSZ was transferred into a mixed conductor in the close vicinity of a Pt electrode by applying strong cathodic polarizations and an oxygen incorporation mechanism including lateral electron transport in YSZ (similar to the situation on GDC here) could be verified by electrochemical and tracer based methods.

For an in-depth understanding of the exact mechanism on the GDC anodes in the present study an exact quantification of the oxygen chemical potential is necessary, which is rather non-trivial. First, we do not know the exact ohmic polarization of the electrolyte. Due to frequency-dependent current paths, the high-frequency intercept in impedance spectra does not represent the electrolyte resistance in dc conditions. Second, the decay of this non-equilibrium chemical potential is non-linear and complicated to calculate.

### 3.3 Comparison of polarity and electrode placement

**Fig. 4:**
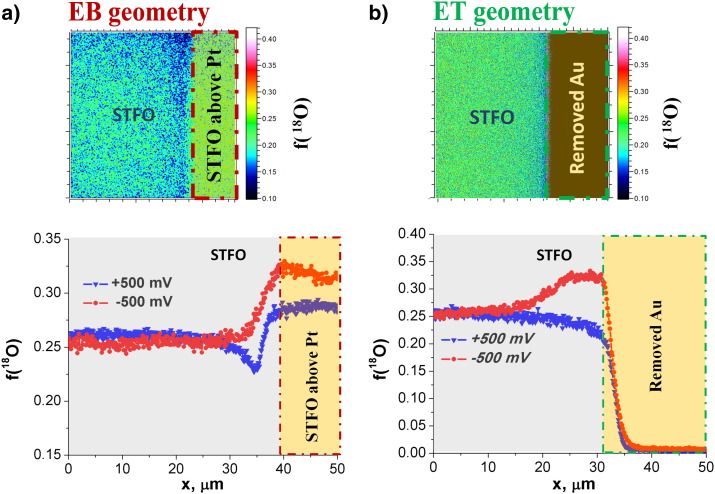
Isotope distribution images and lateral tracer fraction profiles of cathodically (− 500 mV) and anodically (+ 500 mV) polarized noble metal electrodes with (a) EB and (b) ET geometry on the STFO layer.

Enhanced incorporation of ^18^O tracer upon application of cathodic bias could be successfully demonstrated and visualized. Under anodic bias, oxygen release (formation of H_2_O) is promoted and the oxygen incorporation from the tracer gas is reduced in the electrochemically active region. This leads to reduced tracer fraction near the current collector edges, while locations far from the current collector edges exhibit tracer concentrations corresponding to the equilibrium exchange rate. Fig. 4 compares the tracer profiles for + 500 mV and − 500 mV in the ET and EB geometries for STFO. As it was discussed before, the EB geometry leads to an increased isotope concentration on the EB current collector, irrespective of the polarization (Fig. 4a). (The somewhat different isotope concentrations on the current collector are most probably again due to Ar flooding during SIMS measurements and the different analysis area as already discussed above; − 500 mV was analyzed in 150 μm × 150 μm area and + 500 mV in 50 μm × 50 μm area.)

However, comparing the lateral isotope distribution close to the current collector edge clearly indicates reduced ^18^O incorporation with anodic bias (Fig. 4a, EB geometry). For the SIMS measurements with ET geometry the gold current collector was chemically removed after ^18^O exchange. As one can see from Fig. 4b the lateral isotope concentration beneath the ET current collector decays virtually to the natural abundance value. The decay length reflects the in-plane thermal diffusion from the free surface of the MIEC to the MIEC part, which is below the gold layer. Next to the MIEC/current collector edge a cathodic polarization again causes an enhanced ^18^O fraction in a certain width. Under anodic polarization, slightly less ^18^O incorporation is found near the ET current collector. This effect appears less pronounced than in the EB geometry case, due to the impact of the ET current collector discussed above.

## 4 Conclusions

Thin MIEC layers of GDC and STFO on single-crystalline YSZ substrates were exposed to H_2_/H_2_^18^O atmosphere for thermally and electrochemically driven tracer exchange experiments. Rectangular noble metal thin film current collectors were deposited on top and beneath the MIEC layer and used for polarization. The lateral distribution of the tracer revealed several interesting features: (i) In case of thermal tracer exchange, an enhanced tracer fraction is found on top of the metallic current collector due to its ionically blocking nature. At the edges of the current collector, the concentration of ^18^O decreases with a finite step width that is correlated with in-plane diffusion of oxygen ions. (ii) Due to the low electronic conductivity of STFO and GDC, the MIEC area that is influenced by an applied bias is restricted to a region close to the current collector. The width of this active region depends on the bias. It amounts to only 10–15 μm for STFO but more than 100 μm for GDC at a cathodic bias of − 500 mV. (iii) Not only enhanced tracer incorporation due to cathodic bias but also reduced incorporation due to anodic bias could be experimentally resolved in the active region.

## References

[1] Kim S.W., Lee Y., Choi G.M. (2014). Solid State Ionics.

[2] Cho S., Kim Y., Kim J., Manthiram A., Wang H. (2011). Electrochim. Acta.

[3] Jiang J., Shen W., Hertz J.L. (2013). Solid State Ionics.

[4] Cho S., Fowler D.E., Miller E.C., Cronin J.S., Poeppelmeier K.R., Barnett S.A. (2013). Energy Environ. Sci..

[5] Chen X.J., Liu Q.L., Chan S.H., Brandon N.P., Khor K.A. (2007). Fuel Cells Bull..

[6] Tao S., Irvine J.T.S. (2004). J. Electrochem. Soc..

[7] Tao S., Irvine J.T.S. (2003). Nat. Mater..

[8] DeCaluwe S.C., Grass M.E., Zhang C., Gabaly F.E., Bluhm H., Liu Z., Jackson G.S., McDaniel A.H., McCarty K.F., Farrow R.L. (2010). J. Phys. Chem. C.

[9] Chueh W.C., McDaniel A.H., Grass M.E., Hao Y., Jabeen N., Liu Z., Haile S.M., McCarty K.F., Bluhm H., El Gabaly F. (2012). Chem. Mater..

[10] Chueh W.C., Hao Y., Jung W., Haile S.M. (2012). Nat. Mater..

[11] Swaroop S., Kilo M., Kossoy A.E., Lubomirsky I., Riess I. (2008). Solid State Ionics.

[12] Fearn S., Rossiny J.C.H., Kilner J.A., Evans J.R.G. (2012). Solid State Ionics.

[13] Opitz A.K., Lutz A., Kubicek M., Kubel F., Hutter H., Fleig J. (2011). Electrochim. Acta.

[14] Opitz A.K., Kubicek M., Huber Stefanie, Huber Tobias, Holzlechner Gerald, Hutter Herbert, Fleig Jürgen (2013). J. Mater. Res..

[15] Kishimoto H., Sakai N., Yamaji K., Horita T., Brito M.E., Yokokawa H., Amezawa K., Uchimoto Y. (2008). Solid State Ionics.

[16] Yokokawa H. (2012). Solid State Ionics.

[17] Horita T., Yamaji K., Sakai N., Yokokawa H., Kawada T., Kato T. (2000). Solid State Ionics.

[18] Atkinson A., Chater R.J., Rudkin R. (2001). Solid State Ionics.

[19] Baumann F.S., Fleig J., Habermeier H.U., Maier J. (2006). Solid State Ionics.

[20] Nenning A., Opitz A.K., Huber T., Fleig J. (2014). Phys. Chem. Chem. Phys..

[21] Chueh W.C., Lai W., Haile S.M. (2008). Solid State Ionics.

[22] Rothschild A., Menesklou W., Ivers-Tiffée E. (2006). J. Chem. Mater..

[23] Steinvik S., Bugge R., Gjønnes J., Taftø J., Norby T. (1997). J. Phys. Chem. Solids.

[24] Lide D.R., Haynes W.M. (2010). CRC Handbook of Chemistry and Physics.

[25] Holzlechner G., Kubicek M., Hutter H., Fleig J. (2013). J. Anal. At. Spectrom..

[26] Kubicek M., Holzlechner G., Opitz A.K., Larisegger S., Hutter H., Fleig J. (2014). Appl. Surf. Sci..

[27] Peter Velicsanyi, Master's thesis, Vienna University of Technology, 2014.

